# Primary Humidity Standards for Trace Water Measurements in Ultra-High-Purity Process Gases

**DOI:** 10.3390/s26134222

**Published:** 2026-07-03

**Authors:** Vito Fernicola, Giulio Beltramino, Antonio Castrillo, Rugiada Cuccaro, Regina Deschermeier, Volker Ebert, Diana Enescu, Livio Gianfrani, Philipp J. Gliese, Stefania Gravina, Domen Hudoklin, Rezvaneh Nobakht, Isidora Radičević, Lucia Rosso, Shahin Tabandeh

**Affiliations:** 1 INRIM Istituto Nazionale di Ricerca Metrologica, Strada delle Cacce 91, 10135 Torino, Italy; g.beltramino@inrim.it (G.B.); r.cuccaro@inrim.it (R.C.); diana.enescu@valahia.ro (D.E.); r.nobakht@inrim.it (R.N.);; 2 Dipartimento di Matematica e Fisica, Università degli Studi della Campania “Luigi Vanvitelli”, Viale Lincoln 5, 81100 Caserta, Italy; antonio.castrillo@unicampania.it (A.C.); stefania.gravina@unicampania.it (S.G.); 3 Physikalisch-Technische Bundesanstalt (PTB), Bundesallee 100, 38116 Braunschweig, Germany; 4 Department of Mechanical Engineering, Institute of Reactive Flows and Diagnostics, Technische Universität Darmstadt, Karolinenplatz 5, 64289 Darmstadt, Germany; 5 Department of Electronics, Telecommunications and Energy, Valahia University of Targoviste, Strada Aleea Sinaia 13, 130004 Targoviste, Romania; 6 Faculty of Electrical Engineering, University of Ljubljana, Tržaška cesta 25, 1000 Ljubljana, Slovenia; 7 VTT Technical Research Centre of Finland Ltd., Centre for Metrology MIKES, Tekniikantie 1, FI-02150 Espoo, Finland

**Keywords:** ultra-high-purity gases, frost-point temperature, water vapor amount fraction, trace humidity standards, trace water measurement, spectroscopic analyzers

## Abstract

Trace water is one of the most critical matrix contaminants in ultra-high-purity (UHP) process gases, like argon (Ar), nitrogen (N_2_), and many others. Even trace amounts can severely degrade the quality of many products that are reliant on these gases. Despite its importance to advanced technology sectors, notably semiconductor manufacturing, it has proven quite difficult to realize preparative or analytical trace water metrology over the full amount fraction range needed or in the broad spectrum of industrially relevant matrix gases. Within the EU-funded PROMETH2O project consortium, this challenge has been addressed through the development or significant improvement of traceable measurement methods and standards spanning 5 nmol⋅mol^−1^ to 5 µmol⋅mol^−1^, tailored for use in UHP process gas production, such as Ar, N_2_ and clean dry air (CDA). The measurement ranges were extended and the uncertainties were improved while being consistent with the current best practice at primary humidity standard laboratories. The developed standards provide combined standard uncertainties ranging from approximately 0.4 % to 1.5 % in water vapor amount fraction and from 0.03 °C to 0.07 °C in frost-point temperature, while the comb-assisted CRDS system achieves detection limits in the sub-ppb to ppt range. These capabilities were validated in applications that are relevant to process instrumentation and the gas industry. A distributed metrological infrastructure at various European national metrology institutes and partner sites now provides SI-traceable trace water measurements in UHP gases, strongly supporting and extending the calibration capabilities for the gas and semiconductor industries and the associated stakeholders.

## 1. Introduction

Humidity, defined as the amount of water vapor present in gases, exerts a major influence on various chemical, physical, and biological processes. Accurate humidity control is crucial across numerous industries and technologies as moisture levels significantly impact production costs, product quality and performance, safety standards, human health, and working environment conditions. Depending on the application, gas purity requirements range from high-purity gases—typically exceeding 99.999 % purity (grade 5.0), with the total impurity amount fraction below 10 µmol·mol^−1^—to ultra-high-purity (UHP) gases exceeding 99.9999 % purity (grade 6.0 or higher), with the total impurity amount fraction in the nmol·mol^−1^ (ppb) or sub-ppb range. Various high-technology sectors utilize UHP bulk and specialty gases. Bulk gases (e.g., N_2_, O_2_, He, Ar, and H_2_) are primarily used for blanketing, purging, and maintaining ultra-clean environments. Specialty gases (e.g., ammonia (NH_3_) or hydrogen chloride (HCl)) are chemically reactive and utilized in smaller quantities for specific manufacturing processes, such as film deposition or doping.

UHP gases must maintain extremely low contaminant levels to prevent product or process degradation or contamination-related issues [1]. In this context, impurities denote any chemical substances that are present in trace amounts other than the principal gas component, whereas a contaminant is specifically defined as an undesired substance that negatively affects the target process or a process-related measurement. Therefore, while all contaminants are impurities, not all impurities are harmful contaminants. Among all the potential contaminants in UHP gases, water vapor is considered to be one of the most critical due to its strong polarity and its tendency to adsorb onto metallic surfaces or into porous matrices [1]. Adsorption, in this context, refers to the accumulation of water vapor as a surface layer on a given material; the complementary process, desorption, releases bound molecules back into the gas phase and is equally significant for metrological applications at the ppb level.

Water vapor can persist within gas distribution systems, such as stainless-steel pipelines, valves, and pressure regulators, even after extensive purging with dry inert gases like N_2_ and Ar. More elevated water vapor levels can induce corrosion in gas delivery lines and adversely affect processing equipment, particularly when present in reactive gas mixtures such as hydrogen bromide (HBr) or anhydrous hydrogen chloride (HCl). In such environments, water vapor may react to form highly corrosive acidic surface layers that are prone to damage both gas lines and process equipment [1].

Trace humidity in gases is conventionally specified as a water vapor amount fraction (*x*_w_) not exceeding 1 µmol·mol^−1^ (1 ppm), corresponding to a frost-point temperature below −75 °C. This threshold is consistent with the purity requirements specified in SEMI C3 for electronic specialty gases [2]. For frost-point temperature measurements within the trace humidity range, various types of hygrometers are employed, including chilled-mirror hygrometers or laser spectroscopic gas analyzers [3,4,5,6,7]. Less expensive coulometric or metal–oxide sensors are also available [8,9].

The primary metrology on the generation of trace amounts of water vapor is challenging. In fact, high-accuracy metrological studies in the trace water range are rare and largely focused on N_2_ as matrix gas to avoid interaction of matrix gas components at low temperatures. The most relevant standards in the low range are discussed, e.g., in [10,11,12,13,14,15,16,17,18], while relevant metrological comparisons in the trace water regime are reported, e.g., in [19,20,21,22,23].

This paper presents recent advances in trace water generation and measurement in UHP process gases. It focuses on the metrological traceability and the realization of primary and reference humidity standards operating in the range from micromole per mole (ppm) down to nanomole per mole (ppb). The paper is organized as follows. Section 2 presents trace water measurement techniques. Section 3 describes the primary and reference humidity standards developed within the PROMETH2O project, including saturation-based generators from three European national metrology institutes (NMIs) and a coulometric generator. For the coulometric generator of the PTB, the development of the second generation system is described, while its validation is currently in progress and will be reported separately. A comparison of the performance of primary humidity standards developed in other NMIs is also reported in Section 3, as well as the description of a comb-locked cavity ring-down spectrometer. Section 4 presents the validation results and uncertainty budgets for such primary systems. Section 5 discusses the results and identifies future directions.

## 2. Measurement Techniques for Trace Water in UHP Gases

Accurate measurement of water vapor amount fractions in gases, particularly in the ppm to ppb range, presents a significant analytical challenge [24]. Reactive and corrosive gas matrices exacerbate this challenge by reducing sensor stability and sensitivity, slow response times, high measurement drift, hysteresis, and, in severe cases, instrument failure, e.g., by corrosion [25,26]. No single analytical technique universally satisfies all application requirements; each method presents a special combination among sensitivity, complexity, cost, robustness and response time [1,27]. Trace humidity may be quantified by a range of analytical methods, including gas chromatography (GC), mass spectrometry (MS), optical spectroscopy, and sensor-based techniques classified by sensing material and measured quantity; furthermore, sensors are often calibrated with respect to dew- or frost-point temperature even when the measurement principles and the measured quantity are different. The following subsections focus on the methods that are most relevant to UHP gas applications: chilled-mirror hygrometry and optical spectroscopy.

### 2.1. Chilled-Mirror Dew/Frost-Point Hygrometry

Chilled-mirror dew/frost-point hygrometers (CMHs) are the primary reference instruments, measuring the thermodynamic quantity directly, corresponding to the thermodynamic equilibrium between gas-phase water vapor and the condensed phase. This thermodynamic measurand confers direct SI traceability via the temperature scale. CMH instruments are consequently employed by NMIs as transfer standards for humidity calibrations. They operate by cooling a polished metal mirror coated with a gold or rhodium coating by using a Peltier-based thermoelectric cooler (TEC) until condensation occurs. An integrated optical system detects condensation via a reduction in reflected light intensity, while an embedded platinum resistance thermometer (PRT) records the mirror temperature. CMHs operate over approximately −110 °C to 95 °C dew/frost-point temperature, corresponding to a water vapor amount fraction range from 10 nmol·mol^−1^ to approximately 0.85 mol·mol^−1^, often with uncertainties below 0.1 °C [28,29]. Measurement uncertainty can be worsened by co-condensation of contaminant species as well as by Raoult or Kelvin effects from soluble or insoluble mirror deposits [30]. PRT self-heating and an inherently slow response time, particularly at low humidities, limit their suitability in industrial applications [17,31].

### 2.2. Optical Absorption Spectroscopy Methods

Optical absorption methods, such as Fourier transform infrared (FTIR) spectroscopy, enable simultaneous identification and quantification of multiple gas-phase species, including water vapor, down to low ppb levels [32]. Good selectivity for water vapor is achieved at spectral resolutions between 2 cm^−1^ and 4 cm^−1^, while sensitivity and detection limits can be scaled with absorption path length from a few millimeters to a few hundred meters. Detection limits between 10 ppb and 30 ppb are commonly achieved [33]. Below 10 ppb in reactive matrices, laser-based techniques are preferred owing to their higher selectivity and better sensitivity [27].

Tunable diode laser absorption spectroscopy (TDLAS) provides highly selective and sensitive species measurements through targeted high-resolution molecular ro-vibrational absorption lines. The narrow laser linewidth confers greater selectivity when compared to FTIR, and the technique is amenable to fiber-optic integration [34,35,36]. Water vapor detection limits in the ppb range have been demonstrated in inert gases. TDLAS has also been successfully deployed across reactive and corrosive matrices [27,37,38]. In the semiconductor industry, where moisture in UHP bulk gases must remain below 10 ppb to prevent fabrication defects, TDLAS has been widely adopted as a complement to electrochemical sensors, which are susceptible to drift and aging [38].

A first-principles variant of TDLAS, known as dTDLAS, is highly relevant for metrological hygrometry as it allows absolute traceable water vapor quantification without the need for instrument calibration, which is why this approach is also called an optically defined gas standard (OGS). One of the key elements for the realization of an OGS is high-accuracy data for H_2_O molecular line strength [39] and H_2_O collisional broadening [40]. dTDLAS-based water vapor OGSs have been successfully demonstrated even for demanding airborne research conditions [41,42]. The calibration-free property was metrologically validated via comparison against the PTB’s thermodynamic humidity standard [43,44] and later validated between 10 µmol⋅mol^−1^ and 350 µmol⋅mol^−1^ via comparison with the PTB’s first-generation coulometric trace water generator, CTHG-1 [45]. In the CTHG validation the dTDLAS-based H_2_O-OGS showed a relative standard uncertainty of 1.2 % and a precision of 23 nmol⋅mol^−1^ at 2 s time resolution. The OGS concept was successfully transferred to the CRDS technique [46].

Among optical methods, laser-based cavity ring-down spectroscopy (CRDS) is one with the highest sensitivity capable of quantifying trace gases at ppb or sub-ppb (ppt) levels by measuring the decay time of a light pulse within a high-finesse optical cavity [16,47,48,49]. For a comprehensive and up-to-date review on trace-gas detection based on CRDS, the reader can refer to [50]. Cavity enhancement enables effective path lengths up to tens of kilometers, which is not achievable even by multi-reflection FTIR and TDLAS cells. For this reason, CRDS was another important spectroscopic method of choice within PROMETH2O; the comb-locked CRDS system developed at the University of Campania “Luigi Vanvitelli” for SI-traceable trace water detection in UHP gases is described in Section 3.3. Table 1 summarizes and compares key performance indicators for optical trace water vapor measurement methods in UHP process gases.

## 3. Realization of Primary Trace Humidity Standards

The demand for traceable trace humidity measurements has prompted NMIs to develop primary generators and associated metrological infrastructure to underpin the calibration of trace moisture sensors and analyzers in UHP gas applications.

This section describes the primary humidity generators developed within PROMETH2O and a primary measurement method based on CRDS. Three thermodynamic generators based on ice saturation and one coulometric generator collectively cover water vapor amount fractions from a few nanomoles per mole (ppb) to several micromoles per mole (ppm) in N_2_, CDA, and Ar matrices. These systems encompass multiple traceability approaches, including thermodynamic saturation and electrochemical and gravimetric principles. Among the saturation-based methods, two primary thermodynamic configurations are predominantly used to generate traceable reference gas mixtures.

### 3.1. Saturation-Based Humidity Generators

- Single-temperature single-pressure (1T–1P) principle. In a 1T–1P system, a carrier gas is fully saturated with water vapor at a controlled thermodynamic state, defined by a constant saturation temperature (*T*_s_) and pressure (*p*_s_) typically near atmospheric pressure. Following saturation, the gas stream is directly delivered to the devices under calibration. If the pressure drop between the saturator and the point of use is negligible, then *T*_s_ ≅ *T*_fp_.
- Single-temperature two-pressure (1T–2P) principle. In the 1T–2P system, a carrier gas is fully saturated with water vapor at a rigorously controlled thermodynamic state, defined by a constant saturation temperature and pressure (*p*_s_). Following saturation, the gas stream undergoes an isothermal expansion to a lower pressure (*p*_c_), typically near atmospheric conditions. Because the amount fraction of water vapor *x*_w_ is conserved during expansion, the output frost-point temperature is a function of the pressure ratio and the saturation vapor pressure.

Table 2 summarizes the main performance characteristics and technical capabilities of the humidity generators developed and validated by various metrological institutes during the project PROMETH2O and compares such facilities against humidity standard systems developed by other major NMIs. The table presents technical details including working principles, carrier gases, operating ranges, and associated measurement uncertainties.

#### 3.1.1. INRIM Primary Trace Humidity Generator (1T–2P)

The trace humidity generator developed at the INRIM, referred to as INRIM 03 Mark 2, operates in a single-temperature two-pressure (1T–2P) mode. In this configuration the carrier gas, such as N_2_ or Ar, is first saturated at the temperature *T*_s_ and pressure *p*_s_ higher than the atmospheric pressure and then expanded to the point-of-use pressure *p*_c_ slightly higher than the atmospheric pressure (≈1150 hPa). By expanding the humid carrier gas from a saturator pressure as high as 6500 hPa, a frost-point temperature as low as −105 °C is achievable (or, equivalently, a water vapor amount fraction down to 4 nmol·mol^−1^) at the generator outlet, thereby overcoming the frost-point temperature downward limit of the previous version [10]. Under the assumption of mass conservation, the water vapor amount fraction does not change during the expansion; thus, the frost-point temperature of the humid gas *T*_fp_ at the outlet of the generator can be determined using the following relation:

(1) $$\frac{{f\left(p_{s},T_{s}\right)\cdot e}_{s}(T_{s})}{p_{s}}=\frac{{f\left(p_{c},T_{fp}\right)\cdot e}_{s}(T_{fp})}{p_{c}}$$

where the left-hand member of the equation is the amount fraction of water vapor, *x*_w_, calculated from the known variables *p*_s_ and *T*_s_, *p*_c_ is the point-of-use pressure after the expansion, *f*(*p*_c_,*T*_fp_) is the enhancement factor at *T*_fp_ and *p*_c_, while *e*_s_(*T*_fp_) is the saturation water vapor pressure of its pure phase at *T*_fp_. Both enhancement factors were evaluated using the formulation provided by Greenspan [51]. By solving Equation (1) for *e*_s_(*T*_fp_) and then applying Hardy’s formulation [52], the frost-point temperature can be calculated.

**Figure 1:**
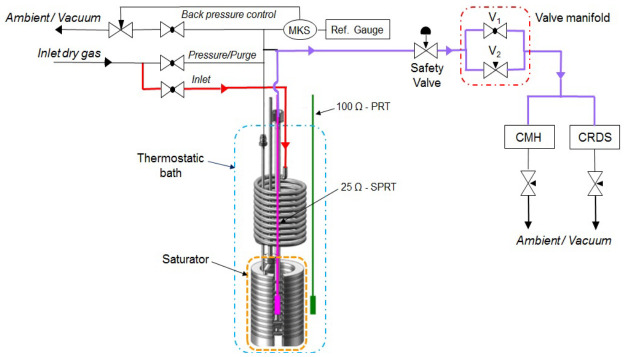
Schematic of the INRIM 03 Mark 2 humidity generator experimental setup. The red line represents the inlet dry gas line; the purple line represents the outlet moist gas line.

The experimental setup of the humidity generator is shown in Figure 1.

The core of the humidity generator consists of an isothermal saturator partially filled with ice, hosted in a thermostatic bath, whose temperature *T*_b_ is measured by a 100 Ω platinum resistance thermometer (PRT) immersed in a liquid bath at a depth corresponding to the saturator outlet. A 25 Ω standard platinum resistance thermometer (SPRT) is inserted into the outlet tube of the saturator to accurately determine the saturation temperature *T*_s_ of the humid gas. The saturation pressure *p*_s_ is controlled by a PID-based back-pressure regulator and measured by means of a high-accuracy capacitance manometer (Paroscientific mod. 745–0.7 MPa f.s., Paroscientific, Inc., 4500 148th Ave. N.E., Redmond, WA 98052, USA). The dry gas flow rate $\dot{V}$ is controlled and measured at the inlet of the saturator in the range from 2 L·min^−1^ to 6 L·min^−1^. A valve manifold at the saturator’s outlet enables the generator to operate in either the 1T–1P (by a high-flow diaphragm bypass valve) or 1T–2P mode (by a bellows-sealed metering valve). The point-of-use pressure, *p*_c_, is measured by a high-accuracy barometer (Keller mod. PAA-33X–1200 hPa f.s., KELLER Pressure, Gallerstrasse 122, 8404 Winterthur, Switzerland).

The humid gas flow at the outlet of the generator is split into two lines to simultaneously feed different types of hygrometers or analyzers. The instruments currently in use include:

1. A chilled-mirror hygrometer (PI/MBW mod. SLX, Process Insights Swiss AG, Seminarstrasse 55/57, 5430 Wettingen, Switzerland) for low frost-point temperature measurements from −110 °C to +20 °C;
2. A cavity ring-down spectrometer (Photonics Technology mod. Pure^n^-T H_2_O, Inner Mongolia Photonics Technologies Co., Ltd., Kangbashi District, Ordos City, Inner Mongolia, China) for water vapor amount fraction measurements between 0.2 nmol·mol^−1^ and 5 µmol·mol^−1^.

#### 3.1.2. UL FE Primary Trace Humidity Generator (1T–2P)

**Figure 2:**
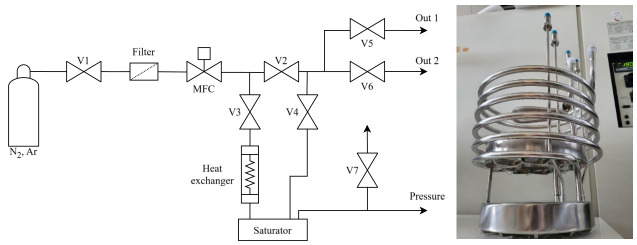
Schematic diagram of the UL FE ultra-low frost-point generator and the stainless-steel high-pressure saturator.

The system operates as a single-pass humidity generator, wherein dry filtered carrier gas (air, N_2_, or Ar of 5-N purity or better) flows through the apparatus a single time prior to being exhausted [53]. As illustrated in Figure 2, the architecture comprises a filter, a mass flow controller (MFC), and a temperature-controlled liquid bath that houses both the heat exchanger and the saturator. Following supplementary purification, the gas flow rate $\dot{V}$ is established by the MFC and directed through the heat exchanger into the saturator (with valves V3 and V4 open and valve V2 closed). The internal saturation path spans approximately 3 m to ensure complete thermodynamic saturation in a single pass [54]. The conditioned gas subsequently exits toward the hygrometer undergoing calibration (via valves V5 and/or V6). During cooling and drying phases, a reverse-flow operation (utilizing open valves V2, V4, and V7) is employed to prevent water adsorption on the outlet tubing, which would otherwise systematically increase the generated moisture content.

The generator supports both single-pressure (1P) and two-pressure (2P) operational modes. In single-pressure (1P) mode, the saturator and the sensor are maintained at nearly equal pressures, exhibiting only a nominal pressure drop [11]. For two-pressure (2P) operation, the gas is initially saturated at an elevated pressure and subsequently expanded isothermally to a lower pressure via an expansion valve located downstream of the saturator. This thermodynamic expansion extends the achievable low frost-point range to temperatures below −100 °C. The core component of the system is the stainless-steel saturator (Figure 2). Its minimum operating temperature is −95 °C, and it has been pressure-tested up to 2 MPa. The saturator must achieve full thermal and phase equilibration of the gas, compensating for the dew-point differential between the inlet and the outlet at sufficient flow rates to supply at least one hygrometer. The 3-m internal fluidic path guarantees efficient saturation [55]. To ensure thermal equilibrium, both the saturator and the heat exchanger are fully immersed in a temperature-stabilized liquid bath [56,57]. The bath temperature is monitored with high accuracy using an SPRT.

#### 3.1.3. VTT Primary Trace Humidity Generator (1T–2P)

The VTT low-frost-point humidity generator (LFPHG) is a thermodynamic saturation- and condensation-based primary humidity generator designed to operate in both single-temperature/single-pressure (1T–1P) and single-temperature/two-pressure (1T–2P) modes [12,58]. This system extends traceable humidity generation down to frost-point temperatures of −100 °C and accommodates operational pressures up to 0.7 MPa.

The VTT generator operates on the 1T–2P thermodynamic saturation principle as described in Section 3.1.1. Carrier gas is saturated at elevated pressure *p*_s_ and temperature *T*_s_, then expanded isothermally to point-of-use pressure pc; the output frost-point temperature and water vapor amount fraction are determined by Equation (1). The reference measurand, expressed as either *T*_fp_ or *x*_w_, is determined by *T*_s_, *p*_s_, and *p*_c_ through Equation (1), and SI traceability is established through calibrated temperature and pressure measurements combined with this validated thermodynamic model.

**Figure 3:**
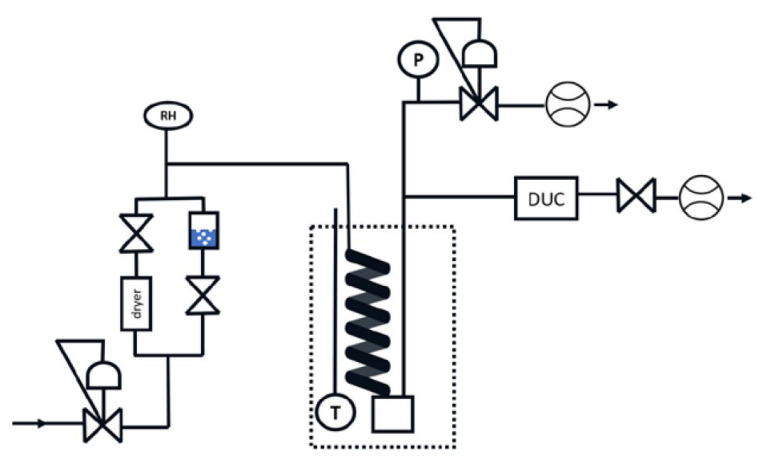
Schematic of the VTT two-pressure low-temperature frost-point generator, where RH denotes a relative humidity hygrometer, T represents the SPRT, P indicates the pressure gauge, and DUC is the device under calibration. The dashed box represents the liquid bath hosting the saturator.

The architecture of the generator (depicted in Figure 3) comprises the following key components: a thermostatically controlled ice saturator/condenser fully immersed in a thermally characterized liquid bath; a calibrated SPRT dedicated to accurate saturation temperature measurement; a back-pressure controller that facilitates system operation at pressures up to 0.7 MPa; a gas purification stage employing a getter dryer to establish a stable base-level humidity; a two-flow conditioning and mixing stage; dedicated outlet branches designed for the connection and calibration of the device under calibration (DUC).

To minimize the effects of incomplete saturation, the saturator design incorporates an extended evaporation path to guarantee thermodynamic equilibrium. Furthermore, thermal stability and spatial temperature homogeneity are continuously monitored using calibrated resistance thermometers. The operational gas pressures at both the saturation stage and the generator outlet are measured using traceably calibrated pressure transducers.

### 3.2. PTB Coulometric Primary Standard (Second-Generation CTHG)

**Figure 4:**
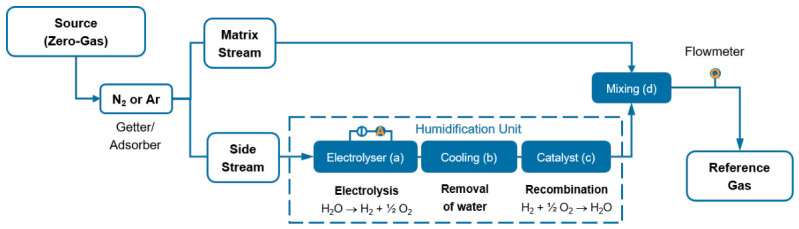
Schematic of the principle of PTB coulometric trace humidity generator (CTHG).

The first-generation coulometric trace humidity generator (CTHG) was developed and operated at the PTB until a few years ago [45]. This generator and its principle were successfully metrologically validated in EURAMET project 1002 [23]. After a gap, the PTB started new actions in the field within the framework of the PROMETH2O project. Within the project, the new second-generation CTHG was designed, set up and brought to operation [13]. Both generators are based on the same principle described in [45]. The CTHG’s core unit typically consists of four main components: an electrolyzer (a), a cooling trap (b), a catalyst acting as the humidification unit (c), and a mixing setup (d). This working principle is illustrated in Figure 4 [13,45].

The core of the humidification unit is formed by the electrolyser (module a) and catalyst (module c). The cooling trap is utilized to condense and remove excess water vapour originating from the electrolysis procedure, which can occur due to saturation when an aqueous electrolyte is employed. The mixing setup enables the generator to operate across a broad water vapor amount fraction range, from 5 nmol⋅mol^−1^ up to 0.04 mol⋅mol^−1^, corresponding to a frost-point temperature range of approximately −100 °C up to −30 °C at ambient pressure.

CTHG operates on the principle of electrochemical hydrolysis according to Faraday’s law, followed by consecutive catalytic recombination to form water. This process encompasses two fundamental steps:

(1) Hydrolysis: $2H_{2}O_{\left(l\right)}\to 2H_{2(g)}+O_{2(g)}$
(2) Catalytic Recombination: $2H_{2(g)}+O_{2(g)}\to 2H_{2}O_{\left(g\right)}$

For an ideal reaction, it is assumed that the amount of water molecules, *n*, is correlated as follows:

(2) $$n_{water,hydrolized(1)}=n_{H_{2(g)}}=n_{water,converted(2)}$$

The amount of hydrolyzed water molecules correlates with the number of transferred electrons and, consequently, the electric charge (*Q*). The subsequent recombination reaction (2) is kinetically controlled and quantitative, ensuring the full conversion of the evolved gases back into water vapor. Consequently, the generated humidity is directly traceable to electrical current. The correlation between the electric current and the resulting amount of water molecules is given by:

(3) $$Q=I\cdot t=F\cdot z\cdot n_{water,converted\left(2\right)}\;↔\;n_{water,converted\left(2\right)}=\;\frac{I\cdot t}{F\cdot z}$$

where *F* = Faraday constant, *z* = number of transferred electrons, *t* = time, *Q* = electric charge, and *I* = electric current.

The humidification unit is embedded within a mixing setup. Inert gases, such as N_2,_ are utilized as the source gas. The inert gas serves as a carrier, ensuring safe generator operation while enabling the generation of low water vapor amount fractions in the reference gas via precise dilution. Regarding operational safety, hydrogen exhibits a lower explosive limit of 4 % and an upper limit of 77 % [59]. To guarantee safe operation, the evolved H_2_ and O_2_ gases must be adequately diluted. Furthermore, the mixing setup is highly beneficial for reaching the lowest trace-humidity range. The mixing ratio *r* is given by Equation (4) [45]:

(4) $$r=\;\frac{m_{w}}{m_{N_{2}}}=\;\frac{M_{w}}{M_{N_{2}}}\;\frac{V_{0}}{\dot{V}}\;\frac{I}{z\cdot F}$$

where $m_{w}$ = mass of water, $m_{N_{2}}$ = mass of nitrogen, $M_{w}$ = molar mass of water, $M_{N_{2}}$ = molar mass of nitrogen, $V_{0}$ = molar volume of ideal gas, and $\dot{V}$ = flow rate of reference gas at 0 °C and 1013.25 hPa.

Equation (5) calculates the amount fraction of water vapor $x_{w}$ obtained from the mixing ratio as follows:

(5) $$x_{w}=\;\frac{r}{\frac{M_{w}}{M_{N_{2}}}+r}+x_{blank}$$

Generators operating on these coulometric principles are cumulative by nature, meaning they add water but cannot actively remove existing humidity from the gas stream. Therefore, the inherent water content of the source gas ($x_{blank}$, e.g., N_2_) must be known and maintained below a specific threshold dictated by the target water vapor amount fraction $x_{w}$.

Since coulometric trace water generators are not based on thermodynamic equilibrium, they provide a complementing technique to validate and compare to other generator types, like saturation-based generators. At the PTB, this type of generator is used to reach very low water vapor amount fractions due to the flexibility and dynamic approach of this system. However, it should be noted that the electrode reactions are sensitive to pressure, temperature, and available reactive surface sites. Stable generator operation, particularly at low frost-point temperatures, therefore, requires stable gas flow and pressure isolation from environmental fluctuations [13]. Thorough characterization of all three modules is required to model generator behavior accurately. Deviations from the ideal reactions are accounted for by applying module-specific efficiency factors determined in preliminary experiments; the associated uncertainty contributions are propagated to the combined measurement uncertainty budget [13].

The experimental validation of the PTB’s second-generation CTHG for calibration services over the theoretical working range (water vapor amount fraction approx. 5 nmol/mol to 0.04 mol/mol) is in progress. At the point of publication, comparisons with traceable CMHs confirm agreement within the combined expanded uncertainty in the range between −30 and −55 °C frost-point temperature (approx. between 0.04 mol/mol and 0.002 mol/mol), which is documented as CMC in the KCDB of BIPM. The validation process for the working range down to −100 °C (water vapor amount fraction approx. 5 nmol/mol) is currently ongoing. The result of the process will be reported in a dedicated publication.

### 3.3. Comb-Locked Cavity Ring-Down Spectrometer (University of Campania)

The principle of operation of CRDS relies on the observation of the temporal evolution of the light coupled in a high-finesse optical cavity. When the injected laser radiation is suddenly turned off, the intracavity field decays exponentially, and the rate of this decay carries direct information about the total optical losses inside the cavity. In an idealized situation, where mirror transmission dominates over other losses, the decay time, *τ*, is determined by the reflectivity of the mirrors and the length of the cavity. When an absorbing molecular species is present inside the cavity, optical losses increase, and the decay time becomes shorter and frequency-dependent. By comparing the decay constant at a given laser frequency with the empty-cavity decay constant, *τ*_0_, the absorption coefficient, *α*, at frequency ν is related to the ring-down times by the equation

(6) $$\alpha \left(\nu \right)=\frac{1}{c}\left(\frac{1}{\tau (\nu )}-\frac{1}{\tau_{0}}\right)$$

where *c* is the vacuum speed of light, and *ν* is the laser frequency. Since it is directly proportional to the number density of the absorbing molecules, CRDS is inherently suited to absolute determinations of trace-gas concentrations. In recent decades, several implementations of CRDS have been proposed, their common focus being the achievement of remarkable sensitivities, which often have been pushed up to the parts-per-trillion (ppt) level [60].

In the case of trace water detection in high-purity gases, the water vapor amount fraction, $x_{w}$, can be determined by using the following expression:

(7) $$x_{w}=\frac{\alpha_{TOT}\cdot k_{B}\cdot T}{S\left(T\right)\cdot p}$$

where *α*_TOT_ is the integrated absorption coefficient, *k*_B_ is the Boltzmann constant, *T* is the thermodynamic temperature, *S*(*T*) is the temperature-dependent line intensity, and *p* is the gas pressure.

The uncertainty of the retrieved molecular concentration critically depends on the determination of the integrated absorption coefficient. The latter, in turn, passes through two important requirements: an absolute, linear, and highly reproducible frequency scale underneath the absorption spectra and the adoption of a refined line-shape model capable of capturing broadening and narrowing signatures of molecular spectra, such as the Doppler and collisional broadening, Dicke narrowing, speed-dependent effects, and possible asymmetries in the absorption profile.

The integration of an optical frequency comb synthesizer (OFCS) into the CRDS setup ensures both absolute frequency determination and long-term spectral stability. The OFCS generates a grid of equally spaced phase-coherent optical modes with highly accurate frequencies, which are established by locking its repetition rate and carrier-envelope offset to a stable reference, namely a GPS-disciplined Rb-clock having a relative drift of the time signal over 1 week of about 3 · 10^−14^. By offset-locking the probe laser to the comb and performing controlled frequency scans, the resulting absorption spectrum gains an absolute SI-traceable frequency axis. This approach enables precise comparisons between spectra recorded over days and allows multi-hour spectral averaging to mitigate low-frequency thermal and mechanical noise, a key factor in achieving record-level sensitivity [61,62]. Furthermore, the vertical scale of the acquired spectra is also fully traceable to SI units. In fact, as shown in Equation (6), the absorption coefficient is directly derived from the measured ring-down time, which is extracted via exponential fitting of decay events. These events are recorded by an acquisition board synchronized to the experiment’s time-base, namely the 10 MHz clock signal provided by the Rb-clock. Other important parameters involved in the water vapor amount fraction determination, such as gas pressure and temperature, are measured with a high-accuracy absolute transducer (MKS, mod. AA06A12TRA, MKS Instruments, Suite 201, 2 Tech Drive, Andover, MA 01810, USA) calibrated at the National Institute of Standards and Technology (NIST) and using a Pt-100 thermometer calibrated at the INRIM, respectively. Finally, retrieving $x_{w}$ requires accurate line intensity factors, *S*(*T*), which are derived from ab initio quantum mechanical calculations recently validated by an international metrological comparison [63]. In the near infrared, for well-characterized water transitions, *S*(*T*) is known to have a relative standard uncertainty ranging from 0.1 % to 1 %, being the specific value used in this work extracted from the HITRAN2024 database [64].

**Figure 5:**
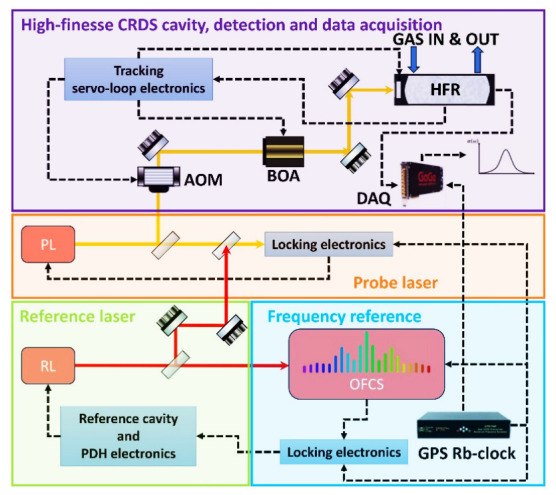
Simplified schematic of the comb-locked CRDS experimental setup. The optical frequency comb synthesizer (OFCS), referenced to a GPS-Rb-clock, provides the absolute frequency scale. The reference laser (RL), actively stabilized to a high-finesse cavity by means of the Pound–Drever–Hall (PDH) method, is locked to the OFCS. The probe laser (PL), phase-locked to the RL, is coupled via an acousto-optic modulator (AOM) and a booster optical amplifier (BOA) into a hemispherical high-finesse resonator (HFR). A 16-bit digitizer (DAQ), whose time-base is disciplined by the Rb-clock, is used for ring-down event acquisitions. Dashed lines represent electrical connections, while continuous lines indicate the laser beams.

The experimental platform, developed at the Ultrasensitive Molecular Spectroscopy Laboratory of the University of Campania, is shown schematically in Figure 5 and described in deep detail in [61,65,66], to which the reader could refer. The SI-traceability chain of the CRDS–OFCS runs as follows: a GPS-disciplined Rb-clock disciplines an optical frequency comb synthesizer (OFCS), which provides an absolute frequency reference to the probe laser; the resulting ring-down time *τ* yields the absorption coefficient α via Equation (6), which, combined with the ab initio calculated line intensity *S*(*T*) [63,64], yields the water vapor amount fraction *x*_w_ via Equation (7). Two tunable external-cavity diode lasers operate at 1.39 μm. One laser acts as reference (RL), being actively stabilized to a high-finesse cavity, which in turn is locked to an OFCS referenced to a GPS-disciplined Rb-clock. The probe laser (PL) is phase-locked to the RL. The PL can be precisely swept across water absorption transitions while maintaining the high spectral purity and stability of the reference laser. A portion of the PL beam is shifted by 40 MHz via an acousto-optic modulator (AOM) and sent into a hemispherical high-finesse resonator, equipped with two spherical mirrors having reflectivity exceeding 99.999 %. Under vacuum conditions, the cavity ring-down time is about 285 ms, corresponding to an effective absorption path length of 170 km. Gas pressure and temperature are monitored with a relative accuracy better than 0.05 %. Constant gas flow conditions are maintained during spectra acquisition to minimize adsorption/desorption effects. Ring-down events are obtained by abruptly shutting off the PL using the AOM, while a booster optical amplifier (BOA) enhances the PL power available at the input of the resonator. The signal transmitted by the high-finesse cavity is detected by means of a low-noise InGaAs photodiode and recorded by a 16-bit digitizer whose time-base is disciplined by the Rb-clock. A LabVIEW program controls the whole experiment.

**Figure 6:**
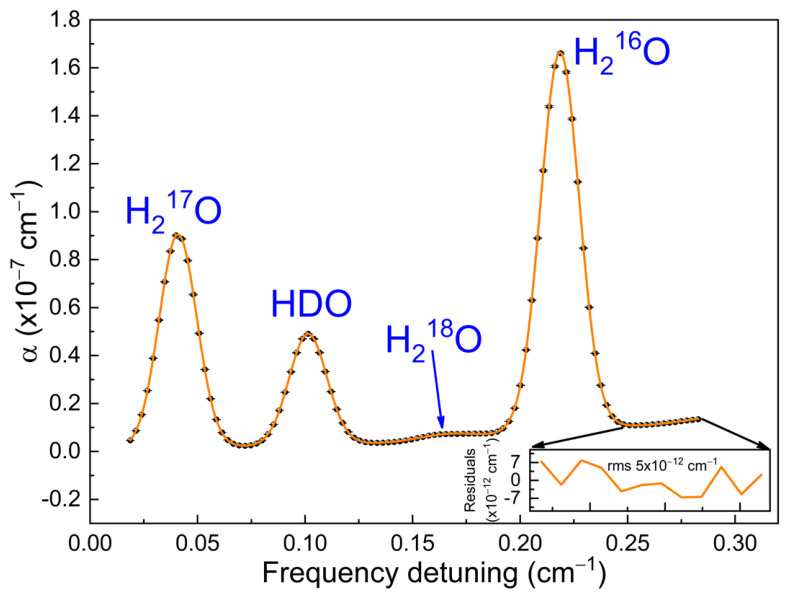
Broadband CRDS absorption spectrum recorded over a frequency span of approximately 9 GHz around 7242 cm^−1^. The spectrum resolves absorption lines from the four most abundant water isotopologues. The gas sample was extracted from the air of the laboratory and maintained under constant flow conditions into the high-finesse cavity at a pressure of about 434 Pa. Black points represent the experimental data, while the orange curve corresponds to a nonlinear least squares fit using a sum of Voigt profiles. The inset panel shows the fit residuals corresponding to the right edge of the spectrum.

To illustrate spectrometer performance, a broadband scan spanning approximately 9 GHz was recorded to simultaneously resolve four absorption features from the most abundant water isotopologues. The resulting spectrum, shown in Figure 6, resolves four absorption features corresponding to the following transitions [64]: the H_2_^17^O 1_1,1_→1_1,0_ component of the ν_1_ + ν_3_ band at 7241.77470 cm^−1^, the HDO 2_2,0_→2_2,1_ component of the 2ν_3_ band at 7241.835537 cm^−1^, the H_2_^18^O 9_4,5_→9_5,4_ component of the 2ν_1_ band at 7241.894981 cm^−1^, and the H_2_^16^O 6_0,6_→5_3,3_ component of the 2ν_1_ band at 7241.951981 cm^−1^ [64].

The spectrum results from the average of 100 consecutive acquisitions; for each acquisition, every spectral point comes from the average of 20 ring-down events. A single spectrum is acquired in about 140 s, with several spectral points and a frequency step of 80 MHz. The measurements were carried out using laboratory air continuously flowing through the measurement cell at a constant pressure of 433.7(1) Pa, while the gas temperature was 23.95(3) °C.

The absorption profile was fitted using a sum of Voigt functions sharing common Doppler widths (properly rescaled by the ratios of the line center frequencies and square root of the mass ratio). The resulting model well reproduces the experimental spectrum and permits identification of the isotopic contributions. For quantitative *x*_w_ retrievals, at the operation pressures, the use of a Voigt line shape model may lead to a systematic uncertainty that is at the percent level. If sub-percent accuracy is required, the use of beyond-Voigt line shapes such as the Hartmann–Tran model [64] is necessary.

The detection of HDO absorption features demonstrates the sensitivity of the spectrometer despite its low natural abundance (~3.1 · 10^−4^). An estimate of the detection sensitivity is obtained from the fit residuals displayed in the inset of Figure 6. Specifically, the minimum detectable absorption coefficient, αmin, that can be determined from the root mean square (rms) of the residuals is equal to 5 · 10^−12^ cm^−1^. Under the measurement conditions described, this corresponds to a limit of detection (LOD) for HDO of approximately 530 ppt. The retrieved *α*_min_ value is consistent with that reported by Castrillo et al. [61] for a comparable integration time. In fact, in [61], the reduction in the LOD by means of spectral averaging was clearly demonstrated. *α*_min_ decreases as *N*^−1/2^ with the number of averaged spectra, indicating uncorrelated (Gaussian) noise. For *N* = 2750, corresponding to approximately two days of averaging time, an αmin of 3.7∙10^−13^ cm^−1^ was achieved.

## 4. Validation of the Primary Humidity Generators

### 4.1. INRIM 03 Mark2 Generator: Validation and Uncertainty

The INRIM trace humidity generator was validated over a frost-point temperature range from −105 °C to −65 °C, corresponding to water vapor amount fractions between 5 nmol·mol^−1^ and 5 µmol·mol^−1^, at gas flow rates between 2 L·min^−1^ and 6 L·min^−1^, as follows:

- By comparing the reference frost-point temperature, *T*_fp,ref_, calculated from the measured saturation temperature *T*_s_, saturation pressure *p*_s_, and point of use *p*_c_ (maintained approximately constant at 1150 hPa), against the frost-point temperature measured by a chilled-mirror hygrometer (PI/MBW SLX);
- By comparing the reference water vapor amount fraction, *x*_w,ref_, calculated from *T*_s_ and *p*_s_, against the amount fraction as measured by CRDS analyzer (Photonics Technologies Pure^n^-T H_2_O).

The validation of the generator was performed by verifying the invariance, within the stated measurement uncertainty, of the difference between the measured and reference values of *T*_fp_ and *x*_w_ while varying pairs of input parameters (*T*_s_, *p*_s_) selected to produce the same nominal frost-point temperature or amount fraction. This approach ensures that the validation remains independent of the (unknown) calibration curve of the comparison instruments. Furthermore, for gas flow rates higher than 2 L·min^−1^, the saturation efficiency of the generator was evaluated at constant *T*_fp_ (and constant *x*_w_).

The differences between the measured and reference frost-point temperatures, evaluated as a function of the saturation pressure *p*_s_ for N_2_ as a carrier gas at a nominal frost-point temperature of −100 °C, remain within the uncertainty bars (*k* = 1), which encompass both the uncertainty of the reference frost-point temperature and the resolution and repeatability of the CMH. Minor pressure-dependent trends are observed but are not statistically significant relative to the associated measurement uncertainties. The same validation procedure was applied across the range from −65 °C to −105 °C. The results confirm that the generator delivers a trace humid gas at the designated frost-point temperature when operating in 1T–2P mode for saturation pressures up to 0.65 MPa. The agreement between measurements at various flow rates confirms saturation efficiency across the 2 L·min^−1^ to 6 L·min^−1^ range.

**Figure 7:**
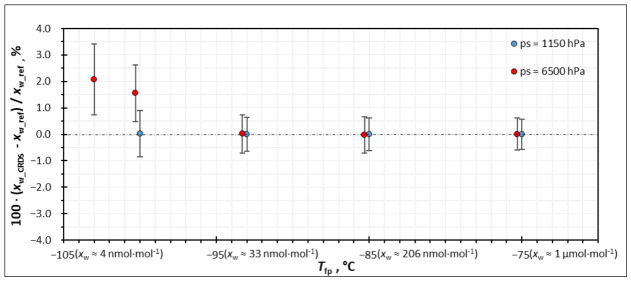
Relative percentage difference between the water vapor amount fraction measured by the CRDS analyzer and the reference value for humid N_2_-H_2_O gas mixture. The plotted results correspond to *p*_s_ = 0.65 MPa and *p*_c_ = 0.115 MPa at each nominal frost-point temperature (and water vapor amount fraction at atmospheric pressure) at a constant flow rate of $\dot{V}$ = 2 L·min^−1^.

**Figure 8:**
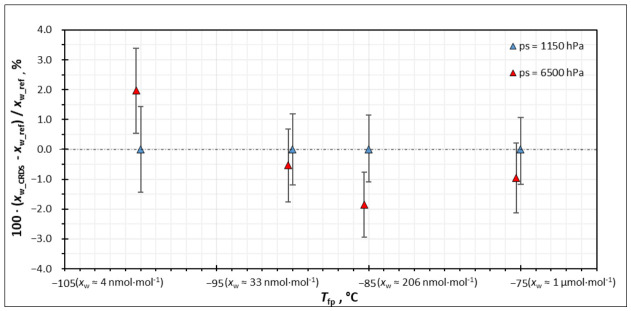
Relative percentage difference between the water vapor amount fraction measured by the CRDS analyzer and the reference value for humid Ar − H_2_O gas mixture. The plotted results correspond to *p*_s_ = 0.65 MPa and *p*_c_ = 0.115 MPa at each nominal frost-point temperature (and water vapor amount fraction at atmospheric pressure) at a constant flow rate of $\dot{V}$ = 2 L·min^−1^.

The relative percent differences between the water vapor amount fraction measured by the CRDS analyzer and the corresponding reference value are presented in Figure 7 and Figure 8 as a function of the nominal frost-point temperature (or amount fraction) for N_2_ and Ar as carrier gases. Measurements carried out at the minimum and maximum saturation pressures (at a constant flow rate of 2 L·min^−1^) demonstrate agreement within their uncertainties for both gas mixtures. This confirms that the generator reliably delivers a humid gas with a known water vapor amount fraction for saturation pressures up to 0.65 MPa. However, definitive conclusions could not be drawn at *T*_fp_ = −105 °C (*x*_w_ ≈ 4 nmol·mol^−1^) due to a lack of available measurements at lower saturation pressures.

An exemplary uncertainty budget for the reference frost-point temperature and water vapor amount fraction is provided in Table 3 for the N_2_-H_2_O gas mixture for a thermodynamic state defined by *p*_s_ = 0.65 MPa, *T*_s_ = −97 °C and *p*_c_ = 0.115 MPa, which corresponds to nominal *T*_fp_ = −105 °C and *x*_w_ = 4 nmol·mol^−1^. The enhancement factor was estimated using the Greenspan moist-air formulation [51], which has been shown to be a good approximation for N_2_ matrices. For Ar–H_2_O mixtures, the use of the Greenspan formulation may underestimate the enhancement factor by up to 0.2 % at high saturation pressures because the H_2_O–Ar cross-virial coefficient differs measurably from that of H_2_O–air. A gas-matrix-specific enhancement factor [11] has been used for the Ar–H_2_O mixture. Once the correct Ar-specific enhancement factor formulation is used, the uncertainty of the formulation itself is of similar magnitude for both N_2_ and Ar. The main contribution to the combined standard uncertainty arises from the uncertainty in the saturation temperature *T*_s_, whereas the enhancement factor on the saturation side *f*(*T*_s_,*p*_s_) contributes marginally to the overall budget *u*_c_(*T*_fp,ref_). Pressure uncertainties were estimated by combining stability, resolution, accuracy and calibration uncertainty of the pressure transducers.

Over the investigated frost-point temperature and water vapor amount fraction ranges, the combined standard uncertainty of *T*_fp,ref_ varies from 0.03 °C for *T*_fp_= −65 °C to 0.07 °C at *T*_fp_ = −105 °C, while the combined standard relative uncertainty of *x*_w,ref_ ranges from 0.4 % at *x*_w_ = 5 µmol·mol^−1^ to 1.5 % at *x*_w_ = 4 nmol·mol^−1^.

### 4.2. UL FE Generator: Validation and Uncertainty

The upgraded frost-point generator was evaluated over a range from −90 °C frost point to +20 °C dew point and pressures from atmospheric to 1 MPa. This corresponds to the enhancement factor experiments of the PROMETH2O project. A key limitation was the performance of the chilled-mirror hygrometer below −80 °C, where a strong dependence on flow and water diffusion through the O-ring seal around the mirror was observed. To address this, experiments were designed to separate the hygrometer flow dependence from the saturator efficiency, and the instrument head was modified to mitigate diffusion through the O-ring.

The reference value depends on several system components, including the dew/frost-point CMH (MBW 373LX, Process Insights Swiss AG, Seminarstrasse 55/57, 5430 Wettingen, Switzerland), SPRT (Accumac Technology, Inc., 90 N William Dillard Drive C-107, Gilbert, AZ 85233, USA), resistance bridge (Batemika mod. UT-One B03A, Batemika d.o.o, Slap 57, 5271 Vipava, Slovenia), and pressure gauge (Druck PACE1000, Baker Hughes, Fir Tree Lane, Groby, LE6 0FH, Leicester, United Kingdom), whose uncertainties were included in the overall uncertainty budget. In addition, the saturator performance with its temperature stability, temperature uniformity, pressure stability, and saturation efficiency significantly contributes to the total uncertainty.

The stability of the saturator temperature was determined from repeated measurements of the liquid-bath temperature, with the maximum standard deviation taken as the stability contribution. Temperature uniformity was evaluated by measurements at multiple positions inside the bath, including two vertical levels and four circumferential points around the saturator.

Saturation efficiency was assessed by varying the gas flow between 0.2 L·min^−1^ and 3 L·min^−1^. Pressure stability in the saturator and at the outlet was derived from repeated pressure measurements, while the pressure drop was calculated as the difference between the saturator pressure and the pressure measured at the output sensor.

The resulting uncertainties for dew-point temperatures between −90 °C and +20 °C are summarized in Table 4.

### 4.3. VTT Generator: Validation and Uncertainty

**Figure 9:**
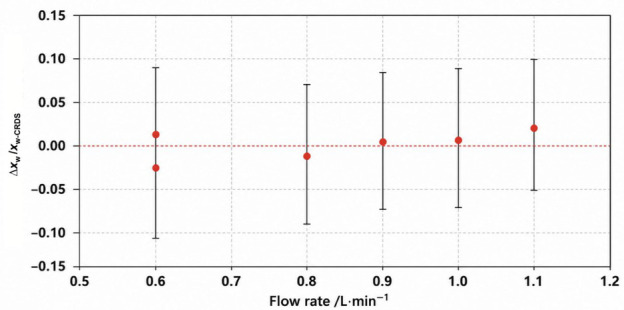
Flow dependence on the condenser, where red circles represent the relative deviation and are calculated as the difference between the water vapor amount fraction at each flow rate and the averaged value of the repeatability experiment. Vertical black error bars indicate the corresponding standard deviations.

The performance of the VTT low-frost-point humidity generator (LFPHG) was validated over its extended operating range with particular focus on ultra-trace humidity levels and elevated pressures. To assess potential incomplete saturation or adsorption effects, a dedicated flow-rate study was performed. The relative deviation of the measured water vapor amount fraction from the mean value of the repeatability experiment is illustrated in Figure 9 as a function of flow rate.

Within the investigated range (0.6 to 1.1) L·min^−1^, no statistically significant systematic flow dependence is observed, as indicated by the trend line passing through the origin, where the deviation is expected to approach zero at infinitesimal flow rates. The deviations remain well within the uncertainty limits, confirming adequate residence time in the saturator and negligible flow-induced bias at the operating flow of 0.7 L·min^−1^ used for low frost-point realization. These results support the assumption of a sufficiently established thermodynamic equilibrium in the saturator under the selected operating conditions within the claimed uncertainty levels.

**Figure 10:**
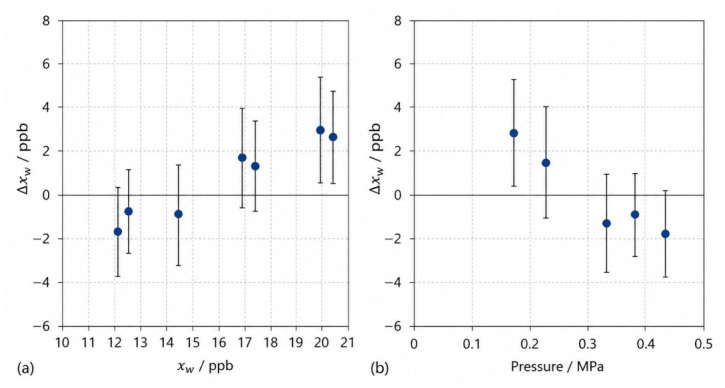
Comparison (difference *Δx*_w_) between the generated reference water vapor amount fraction and the value measured by the CRDS as a function of: (**a**) a range of generated water vapor amount fractions and (**b**) at several saturator pressures. Blue circles represent mean differences, while the vertical black error bars indicate the corresponding standard deviations.

Figure 10 shows the difference between the generated reference water vapor amount fraction and the value measured by a CRDS analyzer, expressed as Δ*x*_w_, as a function of generated amount fraction (Figure 10a) and saturation pressure (Figure 10b).

Across the investigated range, the deviations remain within the calculated expanded uncertainties. No significant systematic trend with water vapor amount fraction is observed in the trace range, and pressure dependence remains consistent with the thermodynamic model. The results confirm the validity of the 1T–2P realization and the proper implementation of the saturation equations introduced in Section 3.1.

The frost-point temperature uncertainty was evaluated using the analytical uncertainty model [67]. An example of the frost-point temperature uncertainty budget at −100.1 °C is shown in Table 5. The water vapor saturation pressure and its uncertainty are based on the IAPWS 2011 formulation [68]. The calculation of the water vapor enhancement factor is also based on Hardy’s equation [52] and its corresponding uncertainty derived from Lovell-Smith’s work [69].

The uncertainty structure confirms that adsorption/desorption and efficiency corrections dominate at ultra-low frost-point temperatures, while pressure-related terms become more relevant at elevated saturation pressures.

## 5. Discussion and Conclusions

### 5.1. Measurement Uncertainty and Metrological Traceability

The primary humidity systems described in this work represent complementary metrological approaches to trace water measurement in UHP process gases. All three thermodynamic generators (the INRIM, the UL FE, and the VTT) established metrological traceability to the SI through calibrated temperature and pressure measurements, combined with the IAPWS formulation for the saturation vapor pressure of ice and well-established enhancement factor formulations based on the work of Greenspan and Lovell-Smith. For the PTB coulometric generator, traceability is routed through electrical current (the ampere) via Faraday’s law.

The expanded uncertainty (*k* = 2) achieved across the thermodynamic generators at frost-point temperatures between −90 °C and −105 °C is consistently below 0.15 °C. These values are competitive with or superior to the best published results from comparable humidity standard generators, as shown in Table 2, and are consistent with the performance requirements of measurement traceability for trace humidity sensors and analyzers in the process gas industry.

The INRIM and the VTT showed that adsorption and desorption effects dominate at very low frost points, where the low vapor density means that even sub-monolayer surface coverage can shift the delivered water vapor amount fraction measurably. This means that any significant improvements would require advances in surface treatment or flow regime control. The INRIM reported the expanded uncertainty at the lowest frost-point temperature (*U*(*k* = 2) = 0.13 °C at −105 °C) with the system operating in 1T–2P mode, while the VTT reported the expanded uncertainty at −100 °C while operating in 1T–1P mode. The UL FE uncertainty at –90 °C (*U*(*k* = 2) = 0.13 °C) reflects the combined contributions of dew/frost-point CMH performance degradation below –80 °C and the modified O-ring sealing mitigation strategy described in Section 4.2.

For the H_2_O–N_2_ system at –97 °C and 6500 hPa, ab initio calculations yield an enhancement factor approximately 0.05 % lower than the Greenspan formulation. For the H_2_O–Ar system, the Greenspan moist-air formulation would introduce a systematic departure of up to 0.2 % to the estimated *x*_w_; this has been corrected by applying the Ar-specific enhancement factor as estimated in [11] via the PROMETH2O *f*-calculator web application [70]. The residual uncertainty of the Ar-specific formulation is comparable to that for N_2_ and is already encompassed in the reported standard uncertainty of *x*_w_ (1.5 % at 4 nmol·mol^−1^).

### 5.2. Operational Range and Gas Matrix Compatibility

The four generators together span a frost-point temperature range from –105 °C (~4 nmol⋅mol^−1^) to +20 °C (~23 mmol·mol^−1^), covering the full operational envelope of UHP process gas qualification from sub-ppb moisture monitoring up to bulk gas characterization. The generators have been validated in N_2_, CDA, and Ar matrices. Validation in H_2_ matrices, which are increasingly critical in the context of hydrogen fuel cell purity specifications (ISO 14687, *x*_w_ ≤ 5 µmol⋅mol^−1^ [71]), remains a priority for future work. The nature of H_2_ introduces additional issues for saturator materials and the enhancement factor formulation, but the PROMETH2O infrastructure provides a validated starting point.

The CRDS–OFCS spectrometer developed at the University of Campania represents an independent primary spectroscopic methodology for the determination of water vapor amount fraction measurement that does not rely on thermodynamic saturation. Its demonstrated LOD of ~530 ppt for HDO with an integration time of about 3.5 h (equivalent to a main isotopologue H_2_^16^O LOD of approximately 100 ppt given the natural HDO/H_2_^16^O ratio of ~3.1 × 10^−4^) surpasses the detection capabilities of all the commercial CRDS analyzers cited in the literature. Moreover, its absolute frequency traceability by exploiting an optical frequency comb synthesizer makes the CRDS–OFCS a potential primary spectroscopic standard. Future work should establish a comparison between the CRDS–OFCS and the thermodynamic generator, which would constitute the first such comparison in Europe at the sub-ppb level.

### 5.3. Validation of the Systems and Present Limitations

The validation approach adopted in this work—confirming the invariance of the difference between a thermodynamic generator’s reference value and a comparison instrument’s indication over a range of input parameter combinations—is independent of the comparison instrument’s absolute calibration. This methodology, applied consistently by the INRIM (Section 4.1) and the VTT (Section 4.3), establishes the internal consistency of each generator over its specified operating range. The agreement between CMH and CRDS analyzer indications and INRIM reference values (Figure 7 and Figure 8) and between the CRDS analyzer and VTT reference values within the stated expanded uncertainties validates the saturation equation implementation of both systems at the level of the comparison instruments’ performance.

An inter-laboratory comparison using a suitable transfer standard would be the next logical step to establish a traceability network at the European level. Until such exercises are available, the goal of European-wide consistency remains provisional.

### 5.4. Conclusions and Future Directions

This paper has described the development, characterization, and validation of four complementary primary and reference trace humidity generators—three thermodynamic (1T–2P) and one coulometric—operating in the range from 5 nmol·mol^−1^ to 5 µmol·mol^−1^ in N_2_, CDA, and Ar matrices.

The expanded uncertainty (*k* = 2) achieved across the three thermodynamic generators at frost-point temperatures between −90 °C and −105 °C is consistently below 0.15 °C, extending previous capabilities. The CRDS–OFCS spectrometer provides an independent SI-traceable spectroscopic determination of *x*_w_ with LOD ~100 ppt (with an integration time of about 3.5 h), establishing a capability not previously demonstrated in a metrology context.

The validation of the PTB’s coulometric second-generation humidity generator (Section 3.2) over the full scope of operation is currently ongoing as part of a consecutive process. A preliminary comparison confirmed the agreement between CTHG and a traceable CMH. A rigorous uncertainty budget and report of the completed validation process of the PTB’s system will be presented in a separate publication.

The future work priorities arising from this program are an inter-laboratory comparison of the generators using a suitable stable transfer standard and the extension of validation to the H_2_ matrix in view of hydrogen fuel cell application requirements.

## Figures and Tables

**Table 1:** Comparison of key metrological performance indicators for optical trace water vapor measurement methods in UHP process gases. LOD and uncertainty values are indicative and depend on integration time, optical path length, and matrix gas. *U* (*k* = 2) values for commercial instruments reflect typical performance.

Method	Typical LOD	Dynamic Range	Linearity	Uncertainty *U* (*k* = 2)	Response Time	References
FTIR	(10–30) nmol⋅mol^−1^	LOD to 100 µmol⋅mol^−1^	Good (Beer–Lambert, 2–3 decades)	5 % to 15 % (calibration-dependent)	1–to-10 min	[32,33]
TDLAS/dTDLAS	(1–10) nmol⋅mol^−1^	LOD to 500 µmol⋅mol^−1^	Excellent (3–4 decades)	1 % to 5 % (calibrated); ~1.2 % at 23 nmol mol^−1^ (dTDLAS)	1–to-10 s	[35,36,44]
CRDS (commercial)	(0.2–1) nmol⋅mol^−1^	LOD to 5 µmol⋅mol^−1^	Excellent (3–4 decades)	5 % to 10 % (calibration-dependent)	1–to-30 s	[47,48,49]
CRDS–OFCS (this work)	0.1 nmol⋅mol^−1^ H_2_^16^O (3.5 h integration)	0.1 nmol⋅mol^−1^ to 5 µmol⋅mol^−1^	Excellent (intrinsic to ring-down principle)	1.5 % at 4 nmol⋅mol^−1^	140 s/spectrum; hours for sub-ppt averaging	This work

**Table 2:** Summary of primary humidity generators for trace water measurement in pure gases developed in this work and comparison with humidity standards available from major NMIs.

NMI	Designator	Working Principle	Carrier Gas	Operating Range	Measurement Uncertainty (*k* = 2)	Reference
PROMETH2O generators (this work)						
INRIM (Italy)	INRIM 03 Mark 2	1T–2P thermodynamic saturation	N_2_, CDA, Ar	(−105 to 0) °C	(0.04 to 0.14) °C	[10]; this work
UL FE (Slovenia)	UL FE 1T–2P	1T–2P thermodynamic saturation	N_2_, CDA, Ar	(−95 to 20) °C	(0.04 to 0.13) °C	[11]; this work
VTT MIKES (Finland)	LFPHG	1T–2P thermodynamic saturation	N_2_, CDA, Ar	(−100 to 0) °C	(0.04 to 0.12) °C	[12]; this work
PTB (Germany)	CTHG (coulometric)	Electrolysis + stream mixing	N_2_, Ar	5 nmol·mol^−1^ to 0.4 mol·mol^−1^	depending on the range	[13]; this work
External NMI generators (published data)						
NIST (USA)	HHG	Two-pressure + divided-flow	N_2_, CDA	(−70 to 85) °C	(0.05 to 0.15 °C (trace range)	[14]
KRISS (Korea)	LFPG 2	2T–2P thermodynamic saturation	N_2_	(7 to 1000) nmol·mol^−1^	(0.33–9.9) nmol·mol^−1^	[15]
NMIJ (Japan)	MSB/DTG	Gravimetric diffusion tube	N_2_	12 nmol·mol^−1^ to 1.4 µmol·mol^−1^	(0.75 to 6.9) %	[16]
NPL (UK)	LFPG	1T–2P thermodynamic saturation	N_2_, CDA	(−90 to 95) °C	(0.05 to 0.15) °C	[17]
METAS (Switzerland)	—	1T–2P thermodynamic saturation	N_2_, CDA	(−90 to +60) °C	(0.05–0.15) °C	[18]

**Table 3:** Uncertainty budget for the water vapor amount fraction and the frost-point temperature of the generated N_2_-H_2_O gas mixture evaluated at *p*_s_ = 0.65 MPa, *T*_s_ = −97 °C and *p*_c_ = 0.115 MPa corresponding to a nominal amount fraction of 4 nmol·mol^−1^ and a nominal frost-point temperature of −105 °C. PDF stands for probability density function.

**Conditions: *T*_fp_ = −105 °C, *p*_s_ = 6500 hPa, *T*_s_ = −97 °C, *p*_c =_ 1150 hPa, *x*_w_ = 4 nmol·mol^−1^**				
**Uncertainty budget for *x*_w,ref_/mol·mol^−1^**				
Source of uncertainty	Standard uncertainty	PDF	Sensitivity coefficient	Contribution to standard uncertainty/mol·mol^−1^
Saturation pure water vapor pressure, *e*(*T*_s_)	0.0000070 Pa	Normal	1.65 · 10^−6^	1.15 · 10^−11^
Enhancement factor on saturation side, *f*(*T*_s_,*p*_s_)	0.0048	Normal	3.90 · 10^−9^	1.89 · 10^−11^
Saturation temperature, *T*_s_	0.069 °C	Normal	7.98 · 10^−10^	5.51 · 10^−11^
Saturation pressure, *p*_s_	48.4 Pa	Normal	6.41 · 10^−15^	3.10 · 10^−13^
**Combined standard uncertainty, *u*_c_(*x*_w,ref_)/mol·mol^−1^**				**5.94 · 10^−11^**
Combined standard uncertainty, *u*_c_(*x*_w,ref_)/pmol·mol^−1^				59
**Uncertainty budget for *T*_fp,ref_/°C**				
Source of uncertainty	Standard uncertainty	PDF	Sensitivity coefficient	Contribution to standard uncertainty/°C
Saturation temperature, *T*_s_	0.069 °C	Normal	8.84 · 10^−1^	6.10 · 10^−2^
Saturation pressure, *p*_s_	48.4 Pa	Normal	6.71 · 10^−6^	3.25 · 10^−4^
Saturation pure water vapor pressure, *e*(*T*_s_)	0.0000070 Pa	Normal	1.84 · 10^3^	1.28 · 10^−2^
Enhancement factor on saturation side, *f*(*T*_s_,*p*_s_)	0.0048	Normal	4.32	2.09 · 10^−2^
Point-of-use pressure, *p*_c_	85.7 Pa	Normal	3.95 · 10^−5^	3.38 · 10^−3^
Enhancement factor at the point of use, *f*(*T*_fp_,*p*_c_)	0.00082	Normal	4.56	3.72 · 10^−3^
**Combined standard uncertainty, *u*_c_(*T*_fp,ref_)/°C**				**0.070**

**Table 4:** Uncertainty budget at different dew/frost-point temperatures over the UL FE generator operating range.

Dew/frost-point temperature/°C	−90	−80	−60	−30	+20
Expanded uncertainty (*k* = 2)/°C	0.130	0.068	0.047	0.039	0.038

**Table 5:** Example of uncertainty budget for frost-point temperature at *T*_fp_ = −100.1 °C.

**Source of Uncertainty**	**Standard Uncertainty/°C**	**PDF**	**Sensitivity Coefficient**	**Contribution to Standard Uncertainty/°C**
Saturation temperature stability, *T*_sat_	0.0020	Normal	1	2.0 · 10^−3^
Saturation temperature uniformity, *T*_bath_	0.0024	Rectangular	1	2.4 · 10^−3^
Calibration uncertainty of the thermometer	0.020	Normal	1	2.0 · 10^−2^
Resolution of thermometer	0.00029	Rectangular	1	2.9 · 10^−4^
SPRT drift	0.00087	Rectangular	1	8.7 · 10^−4^
Self-heating SPRT	0.0022	Rectangular	1	2.2 · 10^−3^
Adsorption/desorption	0.0256	Asy.rectangular	1	2.6 · 10^−2^
Saturation efficiency	0.0043	Rectangular	1	4.3 · 10^−3^
**Combined standard uncertainty, *u*_c_(*T*_fp_)/°C**				**0.033**

## Data Availability

The raw data supporting the conclusions of this article will be made available by the authors on request.

## Nomenclature

AOM	Acoustic–optic modulator
B	Second cross-virial coefficient, cm^3^·mol^−1^
BOA	Booster optical amplifier
*c*	Vacuum speed of light, m·s^−1^
CDA	Clean dry air
CMH	Chilled-mirror hygrometer
CRDS	Cavity ring-down spectroscopy
CTHG	Coulometric trace-humidity generator
dTDLAS	Direct tunable diode laser absorption spectroscopy
DUC	Device under calibration
*e*_s_	Saturation water vapor pressure, Pa
F	Faraday constant, $C\cdot {mol}^{-1}$
FTIR	Fourier transform infrared
*f*	Enhancement factor, dimensionless
GC	Gas chromatography
GPS	GPS-disciplined Rb-clock
HBr	Hydrogen bromide
HCl	Hydrogen chloride
HDO	Water isotopologue H^16^OD
HFR	High-finesse optical resonator
H_2_	Hydrogen
I	Electric current, A
IAPWS	International Association for the Properties of Water and Steam
*k*	Expanded uncertainty, °C
*k*_B_	Boltzmann constant, J·K^−1^
LFPHG	Low-frost-point humidity generator
LOD	Limit of detection
$M_{N_{2}}$	Molar mass of nitrogen, $g\cdot {mol}^{-1}$
$M_{w}$	Molar mass of water vapor, $g\cdot {mol}^{-1}$
$m_{N_{2}}$	Mass of nitrogen, g
*m*_w_	Mass of water vapor, g
${\dot{m}}_{N_{2}}$	Mass flow rate of nitrogen, $kg\cdot s^{-1}$
${\dot{m}}_{w}$	Mass flow rate of water vapor, $kg\cdot s^{-1}$
MFC	Mass flow controller
MS	Mass spectrometry
*N*	Comb tooth order
*n*	Amount of water molecules, mol
NH_3_	Ammonia
N_2_	Nitrogen
NMI	National metrology institute
O_2_	Oxygen
OGS	Optical gas standard
P	Pressure gauge
*p*	Gas pressure, Pa
*p*_s_	Saturation pressure, Pa
*p*_c_	Point-of-use pressure, Pa
PDF	Probability density function
PDH	Pound–Drever–Hall
PID	Proportional–integral–derivative
PL	Probe laser
PLL	Phase-locked loop
ppb	Parts per billion
ppm	Parts per million
ppt	Parts per trillion
PRT	Platinum resistance thermometer
Q	Electric charge, C
*r*	Mixing ratio, dimensionless
rms	Root mean square
RH	Relative humidity hygrometer
RL	Reference laser
Rh	Rhodium
SI	International System of Units
*S*(*T*)	Temperature-dependent line intensity, cm·molecule^−1^
SPRT	Standard platinum resistance thermometer
*t*	Time, s
TEC	Thermoelectric cooler
TDLAS	Tunable diode laser absorption spectroscopy
OFCS	Optical frequency comb synthesizer
*T*	Thermodynamic temperature, K
$T_{fp}$	Frost-point temperature, °C
*T*_fp,ref_	Reference frost-point temperature, °C
$T_{s}$	Saturation temperature, °C
$u_{c}$	Combined standard uncertainty, mol·mol^−1^
$U_{c}$	Expanded uncertainty, K
UHP	Ultra-high purity
$V_{0}$	Molar volume of the ideal gas, m^3^·mol^−1^
V	Volume, m^3^
$\dot{V}$	Flow rate at standard conditions, L·min^−1^
V	Valve
$x_{ref}$	Reference water vapor amount fraction, nmol⋅mol^−1^
$x_{w}$	Water vapor amount fraction, nmol⋅mol^−1^
z	Number of transferred electrons, dimensionless
Greek symbols	
*α*_min_	Minimum detectable absorption coefficient, cm^−1^
*α*_TOT_	Integrated absorption coefficient, cm^−1^
ν	Frequency, Hz
τ	Decay time, s
τ _0_	Empty-cavity decay constant, s

## References

[1] Funke H.H., Grissom B.L., McGrew C.E., Raynor M.W. (2003). Techniques for the measurement of trace moisture in high-purity electronic specialty gases. Rev. Sci. Instrum..

[2] SEMI (2017). SEMI C3—Specification for Gases.

[3] Wiederhold P.R. (1997). Water Vapor Measurement: Methods and Instrumentation.

[4] Heard D., Whalley L.K., Brown S.S., Foken T. (2021). Gas Analysers and Laser Techniques. Springer Handbook of Atmospheric Measurements.

[5] Brewer P.J., Kim J.S., Lee S., Tarasova O.A., Viallon J., Flores E., Wielgosz R.I., Shimosaka T., Assonov S., Allison C.E. (2019). Advances in reference materials and measurement techniques for greenhouse gas atmospheric observations. Metrologia.

[6] Wang J., Wang M., Qian F., Bao X., Jia R., Wang C., Yang X., Wang K., Guo X. (2026). Advances in enhancing the sensitivity of TDLAS for water vapor concentration detection—A review. Infrared Phys. Technol..

[7] Alam N., Islam S.S. (2024). Advancements in trace and low humidity sensors technologies using nanomaterials: A review. ACS Appl. Nano Mater..

[8] Alam N., Yin Y. (2025). Design principles for ultra-low humidity sensors: A mechanism-oriented review. J. Mater. Chem. C.

[9] Korotcenkov G., Simonenko N.P., Simonenko E.P., Sysoev V.V., Brinzari V. (2023). Paper-based humidity sensors as promising flexible devices, state of the art, Part 2: Humidity-sensor performances. Nanomaterials.

[10] Cuccaro R., Beltramino G., Rosso L., Nobakht R., Fernicola V. (2024). Assessment of the INRIM trace water generator and analysis of the uncertainty components down to *−*100 °C frost-point temperature. Metrologia.

[11] Radicevic I., Hudoklin D. (2025). Empirical enhancement factors for trace moisture in nitrogen and argon: Bridging measurement principles. Sens. Actuators B Chem..

[12] Sairanen H., Heinonen M., Högström R., Salminen J., Saxholm S., Kajastie H. (2018). Low-pressure and low-temperature dew/frost-point generator. Int. J. Thermophys..

[13] Gliese P.J., Bubser F., Deschermeier R. (2024). Das coulometrische Prinzip als Grundlage eines Spurenfeuchtegenerators [The coulometric principle as the basis of a trace-moisture generator]. Tech. Mess..

[14] Meyer C.W., Miller W.W., Ripple D.C., Scace G.E. (2012). Uncertainty budget for the NIST Hybrid Humidity Generator. Int. J. Thermophys..

[15] Lee S.-W., Woo S.-B., Kim J.C., Jang E.J., Choi I.B. (2021). Development of a new KRISS low frost-point generator with improved uncertainty from 7 nmol·mol^−1^ to 1000 nmol·mol^−1^. Metrologia.

[16] Abe H., Yamada K.M.T. (2011). Performance evaluation of a trace-moisture analyzer based on cavity ring-down spectroscopy: Direct comparison with the NMIJ trace-moisture standard. Sens. Actuators A Phys..

[17] Takeda N., Carroll P., Tsukahara Y., Beardmore S., Bell S., Yamanaka K., Akao S. (2020). Trace moisture measurement in natural gas mixtures with a single calibration for nitrogen background gas. Meas. Sci. Technol..

[18] Wettstein S., Mutter D. (2018). Design and validation of the MBW standard humidity generators. Int. J. Thermophys..

[19] Bell S., Stevens M., Abe H., Benyon R., Bosma R., Fernicola V., Heinonen M., Huang P., Kitano H., Li Z. (2015). Final report on CCT-K6: Comparison of local realisations of dew-point temperature scales in the range −50 °C to +20 °C. Metrologia.

[20] Meyer C., Abe H. (2020). Bilateral key comparison CCT-K6.2 on humidity standards in the frost-point temperature range from −80 °C to −30 °C. Metrologia.

[21] Brewer P.J., Gieseking B., Ferracci V.F., Ward M., van Wijk J., van der Veen A.M.H., Lima A.A., Augusto C.R., Oh S.H., Kim B.M. (2018). International comparison CCQM-K116: 10 μmol⋅mol^−1^ water vapour in nitrogen. Metrologia.

[22] Brewer P.J., Gieseking B., Ferracci V.F., Ward M., Carroll P., Bell S., Hall B. (2020). International pilot study CCQM-P193: 10 μmol⋅mol^−1^ water vapour in nitrogen. Metrologia.

[23] Brewer P.J., Milton M.J.T., Harris P.M., Bell S.A., Stevens M., Scace G., Abe H., Mackrodt P. (2011). EURAMET 1002: International Comparability in Measurements of Trace Water Vapour.

[24] Ma Z., Fei T., Zhang T. (2023). An overview: Sensors for low humidity detection. Sens. Actuators B Chem..

[25] Bell S., Gardiner T., Stevens M., Waterfield K. (2002). Evaluation of Trace Moisture Sensors—Interim Report.

[26] Bell S., Gardiner T., Gee R.M., Stevens M., Waterfield K., Woolley A. An Evaluation of Performance of Trace Moisture Measurement Methods. Proceedings of the 9th International Symposium on Temperature and Thermal Measurements in Industry and Science.

[27] Raynor M.W., Bertness K.A., Cossel K.C., Adler F., Ye J., Geiger W.M., Raynor M.W. (2013). Trace Water Vapor Analysis in Specialty Gases: Sensor and Spectroscopic Approaches. Trace Analysis of Specialty and Electronic Gases.

[28] Margolis S.A., Huang P.H., Hadaruga N.G., Hadaruga D.I. (2019). Water Determination. Encyclopedia of Analytical Science.

[29] Nie J., Liu X. (2024). A review of dew point sensors: Recent advances and future development. Sens. Actuators B Chem..

[30] Poltera Y., Belmonte A., Luo B.P., Jorge T.R., Vömel H., Wienhold F.G., Peter T. (2021). The “Golden Points” of the cryogenic frost point hygrometer: A new method to reduce measurement noise and improve the precision of stratospheric water vapor measurements. Atmos. Meas. Tech..

[31] Tao J., Luo Y., Wang L., Cai H., Sun T., Song J., Liu H., Gu Y. (2016). An ultrahigh-accuracy miniature dew point sensor based on an integrated photonics platform. Sci. Rep..

[32] Funke H.H., Raynor M.W., Yücelen B., Houlding V.H. (2001). Impurities in hydride gases part 1: Investigation of trace moisture in the liquid and vapor phase of ultra-pure ammonia by FTIR spectroscopy. J. Electron. Mater..

[33] Stallard B.R., Rowe R.K., Garcia M.J., Haaland D.M., Espinoza L.H., Niemczyk T.M. (1993). Trace Water Vapor Determination in Corrosive Gases by Infrared Spectroscopy.

[34] Cui X., Dong F., Zhang Z., Xia H., Pang T., Sun P., Wu B., Liu S., Han L., Li Z., Donchev V. (2018). Environmental application of high sensitive gas sensors with tunable diode laser absorption spectroscopy. Green Electronics.

[35] Werle P. (2011). Accuracy and precision of laser spectrometers for trace gas sensing in the presence of optical fringes and atmospheric turbulence. Appl. Phys. B.

[36] Nelson D.D., Shorter J.H., McManus J.B., Zahniser M.S. (2002). Sub-part-per-billion detection of nitric oxide in air using a thermoelectrically cooled mid-infrared quantum cascade laser spectrometer. Appl. Phys. B.

[37] Funke H.H., Yao J.L., Raynor M.W., Wright A.O. (2004). Using tunable diode laser spectroscopy to detect trace moisture in ammonia. Solid State Technol..

[38] McAndrew J., Bartolomey M., Girard J.M., Goltz G., Han J.M. (2000). Implementing on-line and in-situ moisture monitoring in reactive gas environments. Micro-Santa Monica.

[39] Pogány A., Klein A., Ebert V. (2015). Measurement of water vapor line strengths in the 1.4–2.7 μm range by tunable diode laser absorption spectroscopy. J. Quant. Spectrosc. Radiat. Transf..

[40] Nwaboh J.A., Werhahn O., Ebert V. (2021). H_2_O collisional broadening coefficients at 1.37 μm and their temperature dependence: A metrology approach. Appl. Sci..

[41] Buchholz B., Afchine A., Klein A., Schiller C., Krämer M., Ebert V. (2017). HAI, a new airborne, absolute, twin dual-channel, multi-phase TDLAS-hygrometer: Background, design, setup, and first flight data. Atmos. Meas. Tech..

[42] Buchholz B., Ebert V. (2018). Absolute, pressure-dependent validation of a calibration-free, airborne laser hygrometer transfer standard (SEALDH-II) from 5 to 1200 ppmv using a metrological humidity generator. Atmos. Meas. Tech..

[43] Buchholz B., Böse N., Ebert V. (2014). Absolute validation of a diode laser hygrometer via intercomparison with the German national primary water vapor standard. Appl. Phys. B.

[44] Nwaboh J., Pratzler S., Ebert V. (2023). First metrological validation of TwOGaSt, a new, absolute dTDLAS-trace-hygrometer, using the primary, coulometric, trace water vapour generator at PTB. Tech. Mess..

[45] Mackrodt P. (2012). A new attempt on a coulometric trace humidity generator. Int. J. Thermophys..

[46] Pogány A., Lüttschwager N., Banik G.D., Persijn S., de Boed E.J.J., Sutour C., Macé T., Nwaboh J.A., Werhahn O., Ebert V. (2025). Towards an optical gas standard for ammonia measurements in air at ambient levels. J. Quant. Spectrosc. Radiat. Transf..

[47] Abe H., Hashiguchi K., Lisak D., Honda S., Miyake T., Shimizu H. (2021). A miniaturized trace-moisture sensor based on cavity ring-down spectroscopy. Sens. Actuators A Phys..

[48] Abe H., Amano M., Hashiguchi K., Lisak D., Honda S., Miyake T. (2023). Improvement of spectral resolution in a miniaturized trace-moisture sensor using cavity ring-down spectroscopy: Performance evaluation using a trace-moisture standard in He. Sens. Actuators A Phys..

[49] Casado M., Landais A., Stoltmann T., Chaillot J., Daëron M., Prié F., Bordet B., Kassi S. (2024). Reliable water vapour isotopic composition measurements at low humidity using frequency-stabilised cavity ring-down spectroscopy. Atmos. Meas. Tech..

[50] Hu S.M., Chen W., Venables D.S., Sigrist M.W. (2021). Trace gas measurements using cavity ring-down spectroscopy. Advances in Spectroscopic Monitoring of the Atmosphere.

[51] Greenspan L. (1976). Functional equations for the enhancement factors for CO_2_-free moist air. J. Res. Natl. Bur. Stand. A.

[52] Hardy B. (1998). ITS-90 formulation for vapor pressure, frost point temperature, dewpoint temperature, and enhancement factors in the range −100 to +100 °C. Proceedings of the Third International Symposium on Humidity and Moisture, London, UK, 6–8 April 1998.

[53] FitzGerald C., Mac Lochlainn D., Strnad R., Hodžić N. (2020). Development of a new primary humidity measurement standard. Meas. Sci. Technol..

[54] Cuccaro R., Rosso L., Smorgon D., Beltramino G., Tabandeh S., Fernicola V. (2018). Development of a low frost-point generator operating at sub-atmospheric pressure. Meas. Sci. Technol..

[55] Fernicola V., Arpino F. Design and modelling of a low frost-point humidity generator. Proceedings of the Joint International Symposium on Temperature, Humidity, Moisture and Thermal Measurements in Industry and Science (TEMPMEKO/ISHM).

[56] Hudoklin D., Dmovšek J. (2008). The new LMK primary standard for dew-point sensor calibration: Evaluation of the high-range saturator efficiency. Int. J. Thermophys..

[57] Hudoklin D., Jokovski B., Nielsen J., Dmovšek J. (2008). Design and validation of a new primary standard for calibration of the top-end humidity sensors. Measurement.

[58] Sairanen H., Heinonen M., Högström R. (2015). Validation of a calibration set-up for radiosondes to fulfil GRUAN requirements. Meas. Sci. Technol..

[59] Schröder V., Emonts B., Janßen H., Schulze H.-P. (2004). Explosion limits of hydrogen/oxygen mixtures at initial pressures up to 200 bar. Chem. Eng. Technol..

[60] Gianfrani L., Hu S.-M., Ubachs W. (2024). Advances in cavity-enhanced methods for high precision molecular spectroscopy and test of fundamental physics. La Riv. Del. Nuovo C..

[61] Castrillo A., Khan M.A., Fasci E., D’Agostino V., Gravina S., Gianfrani L. (2024). Demonstration of record sensitivity for water vapour detection by means of comb-locked cavity ring-down spectroscopy. Optica.

[62] Kassi S., Campargue A. (2012). Cavity ring-down spectroscopy with 5 × 10^−13^ cm^−1^ sensitivity. J. Chem. Phys..

[63] Hodges J.T., Bielska K., Birk M., Guo R., Li G., Lim J.S., Lisak D., Reed Z.D., Wagner G. (2025). International comparison CCQM-P229 pilot study to measure line intensities of selected ^12^C^16^O transitions. Metrologia.

[64] Gordon I.E., Rothman L.S., Hargreaves R.J., Gomez F.M., Bertin T., Hill C., Kochanov R.V., Tan Y., Wcisło P., Makhnev V.Y. (2026). The HITRAN2024 molecular spectroscopic database. J. Quant. Spectrosc. Radiat. Transf..

[65] Fasci E., D’Agostino V., Khan M.A., Gravina S., Porzio G., Gianfrani L., Castrillo A. (2023). Comb-assisted cavity ring-down spectroscopy for ultra-sensitive traceable measurements of water vapour in ultra-high purity gases. J. Phys. Conf. Ser..

[66] Fasci E., Khan M.A., D’Agostino V., Gravina S., Fernicola V., Gianfrani L., Castrillo A. (2023). Water vapor concentration measurements in high purity gases by means of comb-assisted cavity ring-down spectroscopy. Sens. Actuators A Phys..

[67] BIPM, IEC, IFCC, ILAC, ISO, IUPAC, IUPAP, OIML (2008). JCGM 100:2008; Evaluation of Measurement Data—Guide to the Expression of Uncertainty in Measurement (GUM).

[68] Wagner W., Riethmann T., Feistel R., Harvey A.H. (2011). New equations for the sublimation pressure and melting pressure of H_2_O ice Ih. J. Phys. Chem. Ref. Data.

[69] Lovell-Smith J. (2007). An expression for the uncertainty in the water vapour pressure enhancement factor for moist air. Metrologia.

[70] PROMETH2O *f*-Calculator Web-Application for the Traceability of Thermophysical Properties in Moist Gas Mixtures. https://prometh2o.eu/en/f-calculator.

[71] (2019). Hydrogen Fuel—Product Specification.

